# Multidimensional insights into the repeated electromagnetic field stimulation and biosystems interaction in aging and age-related diseases

**DOI:** 10.1186/s12929-022-00825-y

**Published:** 2022-06-13

**Authors:** Felipe P. Perez, Joseph P. Bandeira, Cristina N. Perez Chumbiauca, Debomoy K. Lahiri, Jorge Morisaki, Maher Rizkalla

**Affiliations:** 1 Indiana University School of Medicine, Indianapolis, IN USA; 2 Division of General Internal Medicine and Geriatrics, Department of Medicine, Indiana University School of Medicine, Indianapolis, IN USA; 3 Division of Rheumatology, Department of Medicine, Indiana University School of Medicine, Indianapolis, IN USA; 4 Department of Psychiatry, Institute of Psychiatric Research, Neuroscience Research Center, Indiana University School of Medicine, Indianapolis, IN USA; 5 Department of Medical and Molecular Genetics, Indiana University School of Medicine, Indianapolis, IN USA; 6 Department of Bioengineering, University of Illinois at Chicago, Chicago, IL USA; 7 Department of Electrical and Computer Engineering, Indiana University-Purdue University, Indianapolis, IN USA

**Keywords:** Repeated electromagnetic field stimulation, Autophagy, Chaperones, HSF1, Amyloid beta, Alzheimer’s disease, Aging, Age-related disease

## Abstract

We provide a multidimensional sequence of events that describe the electromagnetic field (EMF) stimulation and biological system interaction. We describe this process from the quantum to the molecular, cellular, and organismal levels. We hypothesized that the sequence of events of these interactions starts with the oscillatory effect of the repeated electromagnetic stimulation (REMFS). These oscillations affect the interfacial water of an RNA causing changes at the quantum and molecular levels that release protons by quantum tunneling. Then protonation of RNA produces conformational changes that allow it to bind and activate Heat Shock Transcription Factor 1 (HSF1). Activated HSF1 binds to the DNA expressing chaperones that help regulate autophagy and degradation of abnormal proteins. This action helps to prevent and treat diseases such as Alzheimer’s and Parkinson’s disease (PD) by increasing clearance of pathologic proteins. This framework is based on multiple mathematical models, computer simulations, biophysical experiments, and cellular and animal studies. Results of the literature review and our research point towards the capacity of REMFS to manipulate various networks altered in aging (Reale et al. PloS one 9, e104973, 2014), including delay of cellular senescence (Perez et al. 2008, Exp Gerontol 43, 307-316) and reduction in levels of amyloid-β peptides (Aβ) (Perez et al. 2021, Sci Rep 11, 621). Results of these experiments using REMFS at low frequencies can be applied to the treatment of patients with age-related diseases. The use of EMF as a non-invasive therapeutic modality for Alzheimer’s disease, specifically, holds promise. It is also necessary to consider the complicated and interconnected genetic and epigenetic effects of the REMFS-biological system’s interaction while avoiding any possible adverse effects.

## Introduction

### REMFS in current literature

The massive proliferation of EMF devices has awakened great curiosity to understand the mechanism of their interaction with biological systems. Recently, numerous researchers have evaluated this interaction [1–4]. Their results suggest specific conditions of experimental and clinical RF exposure may lead to multi-target effects [5] through activation of several biological pathways [5]. Relevant effects of EMF exposures on the pathways known to be involved in the aging process have been identified by in vitro studies (Table 1) and in vivo studies (Table 2). These studies have looked at specific techniques involving various field strengths and exposure time (dosimetry).

**Table 1:** In vitro experiments

Biological effects	EMF	Bio-system	Field strength	Exposure time	References #
HSF1 activation	60 Hz	Human HL60 cells	0.8µT, 80µT	20 min	[6]
Mitochondrial activity	50 Hz	Human SH-SY5Y	100-μT	24 h	[7]
ROS production	900 MHz	Rat astroglia	10 V/m	5, 10, 20 min	[8]
Blastogenesis	3 Hz	Human lymphocytes	60 G	72 h	[9]
45Ca incorporation	30 Hz	Human lymphocytes	0.15 mT		[10]
Channel-activity	42.25 GHz	Kidney cells	2 mW/cm^2^ continuous	20–30 min	[11]
Voltage‐gated calcium	ELF and Microwave	Human and animal cells	Pulsed and continuous	Seconds to min	[12]
Growth-related	60 Hz	Human lymphoma	10 mV/cm continuous	1 h	[13]
RNA synthesis	60 Hz	HL-60 cells	5.7 µT continuous	20 min	[14]
DNA synthesis	15 Hz to 4 kHz	Human fibroblasts	0.023–5.6 G, sinusoidal	1,2,24 h	[15]
DNA synthesis	75 Hz	Human chondrocytes	2.3 mT pulsed	6 to 30 h	[16]
Expression of microRNA	75 Hz	Mononuclear cell from AD	3 mT pulsed	15, 30, 60 min	[17]
Expression of microRNA	50 Hz	Mouse GC–2 cells	1 mT, 2 mT and 3 mT pulsed	72 h (5 min on/10 min off)	[18]
reduction of oxidative stress	75 Hz	Human SH-SY5Y	2 mT pulsed	10 min, 4 times a week	[19]
reduction of oxidative stress	60 Hz	Human SH-SY5Y	4 to 10 mT continuous	20 min	[20]
expression of hsp70	60 Hz	Human breast cells	3 mT continuous	1 to 3 h	[21]
Cytoprotection	60 Hz	Rodent cardiomyocytes	8μT	30 min	[22]
Ubiquitin–proteasome system	50 Hz	Caco 2 cells	1 mT	24–72 h	[23]
Ubiquitin–proteasome system	1.95 MHz	KB cells	3 mW/g	1, 2, 3 h	[24]
Ubiquitin–proteasome system	100 mT	Rat hippocampal cells	100 mT continuous	15 min	[25]
Autophagy-lysosome systems	75 Hz	SH-SY5Y cells	2 mT pulsed	1 h	[26]
Inflammation	27.12 MHz	Human dermal fibroblasts	591 V/m pulsed	30 min	[27]
Cellular senescence	50 MHz	Mouse fibroblasts	0.5 W/Kg continuous	30 min/d	
β-amyloid (Aβ) deposition	64 MHz	Human neurons	0.4-to 0.9 continuous	1 h/d × 21 days

**Table 2:** In vivo experiments

Biological effects	EMF	Bio-system	Field strength or SAR	Exposure	References #
Oxidative stress	15 μT	Vicia faba L	15 μT	8 h/d × 8 days	[28]
Expression of microRNA	2.4 GHz	Rat brain	2420 μW/kg	24 h/d × 12 months	[29]
Reduction of oxidative stress	10 kHz	Maize	3mT	6 h/d × 4 days	[30]
Reduction of oxidative stress	900 MHz	Rat	0.18 W/kg	1 h/d × 21 days	[31]
Reduction of oxidative stress	60 Hz	Chick embryos	8 μT	20 min	[32]
Inflammation	1–100 Hz	Mice	1–100 Gauss pulsed	30–45 min	[33]
Inflammation	27.12 MHz	Human after breast surgery	591 V/m pulsed	30 min	[34]
Mitochondrial enhancement	918 MHz	AD mice	0.25–1.05 W/kg, pulsed	2 h/d × 1 month	[35]
Neuronal activity	918 MHz	AD mice	0.25–1.05 W/kg, pulsed	2 h/d × 2 months	[36]
β-amyloid (Aβ) deposition	918 MHz	AD mice	0.25–1.05 W/kg, pulsed	2 h/d × 7 to 9 months	[37]
Osteoarthritis	37 and 75 Hz	Guinea pigs	3mT pulsed	6 h/day × 6 months, pulsed	[38]

*SAR* specific absorption rate

The results listed in Tables 1 and 2 demonstrate that the effects of biological mechanisms influenced by REMFS are likely extensive and may act in multiple distinct pathways. These data support possible therapeutic implications of REMFS on the aging process and age-related diseases, such as Late Onset Alzheimer’s disease (LOAD), which is also supported by the results of our prior experiments and theories [39]. In later sections of this paper, we will specify these potential mechanisms.

### The consistencies and inconsistencies between the in vivo and in vitro data

As DNA damage is frequently a prerequisite for cancerous diseases, reviews on this topic provide an experimental body of evidence on the effect of EMF on genetic material. Diab’s studies on the matter showed contradictory data in in vivo and in vitro experiments [40]. Several studies of both in in vivo and in vitro samples showed a detrimental effect on DNA when exposed to EMF. Some reports showed no injury to DNA in either in in vivo or in vitro experiments. Results from other experiments were inconclusive [40]. These conflicting findings were probably caused by variability in the EMF generators, different experimental methods including time of exposure, and characteristics of the specific samples (age, genetic differences, size, tissue penetration, anatomical differences [41], etc.).

It is important to understand that the in vitro results cannot easily be extrapolated to in vivo results. This is because the energy absorbed by an object is dependent on the way the EMF is able to penetrate the object [42]. The physiological characteristics of in vivo specimens differ significantly from that of an in vitro cell, and exposure to the same external field would result in an entirely different internal field. Hence, it becomes important to determine what external fields would produce similar internal fields inside both in vitro and in vivo specimens before we reach any conclusions about the biological effects of a specific EMF frequency or field intensity.

Another concern is tissue penetration, which is inversely proportional to the frequency of the EMF. With in vitro specimens there is no need to calculate tissue penetration, but in vivo samples have complex calculations for tissue penetration due to the presence of multiple tissue layers, the geometry of the tissues, and their specific dielectric properties (conductivity and permittivity). As the frequency is increased, the penetration depths of the tissue layers change such that the largest part of the incident energy may be transmitted at one frequency and absorbed at another. Sufficiently high frequencies should result in a small penetration depth, resulting in superficial penetration.

## REMFS in cell death and senescence

### REMFS delays cell death

In our REMFS initial study, we exposed T-cells and lymphoblasts cultures to a frequency of 50 MHz and a power of 0.5W [43]. We found a 20% reduction (p < 0.05) in LDH over 3 weeks of REMFS treatments. The 30-min REMFS exposures were the most stable between REMFS treatment times, so this was selected as the minimal optimal treatment regimen. We demonstrated a 34% reduction in LDH release in REMFS-treated quiescent T-cells compared to control cells following treatment with 30 min of REMFS for 7 consecutive days (p < 0.01), suggesting a significant protective effect from REMFS. We corroborated the protective effects of REMFS with a Trypan blue exclusion study. Results showed that REMFS decreased T-cell death for 7 days, with maximal benefit using 30 min of daily treatments. These data demonstrated that REMFS of low energy and intensity (50 MHz / 0.5 W) can play a cytoprotective role [43].

### REMFS delays cellular senescence

REMFS treatments produced effects associated with cellular senescence [43]. We treated knockout (KO) and control mouse fibroblasts at 100% of lifespan completed or 23 cell population doubling (CPDL) with REMFS at 50 MHz / 0.5 W every 3 days for 14 days. We found that when cells passed from CPDL 3 to 23, they became larger, vacuolated cells with more diverse morphotypes than cells at earlier CPDL. Interestingly, REMFS reversed and delayed senescent morphology, enlargement, and variation of HSF1 + / + mouse fibroblasts, but not HSF1 −/− mouse fibroblasts. These results emphasize the importance of REMFS effects on HSF1. REMFS treated HSF 1 + / + mouse fibroblasts remained smaller in size and more spindle-shaped with more parallel positioning of the cells. In addition, there were less multinucleated cells. We also observed that REMFS prolonged the replicative lifespan to 29 CPDL of murine fibroblast HSF 1 + / + compared to 23 CDLP in non-REFMS treated fibroblasts [43].

We also compared the CPDL tables of HSF1 + / + and HSF1 knockout mouse fibroblasts in treated vs non-treated cultures [43]. Both groups grew at similar rates until CPDL 18, after which REFMS-exposed fibroblasts CPDL appeared to prevent the decline in cell proliferations rates observed in untreated cells. REMFS-treated fibroblasts demonstrated 138 days of proliferative lifespan, compared to 118 days in non-treated cultures. This represented an increase of 17% in lifespan. Similar to prior experiments, HSF1- knockout cultures did not show response to the treatments, achieving only 23 CPDLs with 100 days of replicative life span.

## REMFS at the organismal level

### REMFS in aging

Several short-term exposure studies have shown that REMFS increases lifespan in mice, worms, and flies. In a recent study, mice had an increased average lifespan when exposed to REMFS with an alternating magnetic field of 100 nT and 60 μT [44]. In another study with rotating Magnetic Field (0.2 T, 4 Hz), REMFS exposure slowed the aging process and prolonged the lifespan of *C. elegans* and of Human Umbilical Vein Endothelial Cells (HUVECs). REMFs also improved activity, reduced pigment accumulation, and delayed paralysis induced by Aβ, as well as increased heat tolerance and oxidative stress resistance [45]. A higher frequency study (10 GHz) extended the life span of *Basc* females fruit fly *Drosophila melanogaster* [46]. In another high frequency (1–10 THz) study, there was no increase in survival in early life but increased survival in later life [47].

An interesting study of *Drosophila melanogaster* lifespan showed that population was decreased or increased depending on parameters of the REMFS exposures [48]. There are, however, many other studies that show that prolonged EMF exposures do not increase lifespan in multiple organisms. This is most likely due to the use of different frequencies, powers, times or cell types [49–52].

### REMFS in Alzheimer’s disease

AD and Lewy body dementia (LBD) usually emerge during aging, when the proteostasis quality control is unable to prevent the aggregation of misfolded proteins. AD is characterized by Aβ peptides, an APP fragment of 39–43-amino acids [53]. Efficacy and safety of REMFS have been demonstrated in Transgenic (Tg) AD mouse models in vivo. An initial REMFS study prevented or reversed memory loss in Tg AD mouse model (AβPPsw) when a pulsed and modulated RF-EMF at 918 MHz with a SAR of 0.25–1.05 W/kg was applied over a 7 to 9 month period [54]. REMFS exposed Tg mice preserved good cognitive function, whereas control Tg mice showed cognitive decline. Tg mice of advanced age (21–27 months) with daily REMFS exposure for 2 months showed improved memory in the Y-maze task, although not in more complex tasks [36]. These older Tg controls showed high levels of Aβ aggregates with treated mice showing a 24–30% decrease of Aβ deposits. These data suggest a degradation of Aβ deposits with REMFS exposure. In addition, these long-term treatments were found to be safe (daily for up to 9 months) without any toxic effects on multiple health parameters, including oxidative stress, brain histology, brain heating, damage to DNA, or cancer in peripheral tissues [55].

A higher frequency study (1950 MHz) showed decreased AD pathology in Tg-5xFAD transgenic mice, which overexpress APP, and wild type (WT) mice treated with REMFS at 1950 MHz with SAR 5 W/kg for 2 h per day, 5 days per week [56]. This long-term exposure to REMFS decreased Aβ plaques, APP, and APP carboxyl-terminal fragments in the brain. REMFS also decreases the expression of β Beta secretase 1 (BACE1) to prevent inflammation.

Additionally, REMFS reverses cognitive decline in AD mice. REMFS treatment showed that when compared to WT mice, 5 genes that are all implicated in Aβ processing (Tshz2, Gm12695, St3gal1, Isx and Tll1), are affected in Tg-5xFAD mice treated with REMFS. Specifically, WT showed the same genetic profile to non-REFMS-treated Tg mice, while REMFS-treated Tg mice demonstrated different patterns. Therefore, these data suggest that chronic REMFS treatment influence Aβ processing in AD mice, but not in wild or Tg controls [56].

Altogether, AD mouse studies and human brain cell studies revealed that REMFS exposures reduce Aβ. It also prevents and decreases brain Aβ aggregation without causing any inflammatory reaction as seen in passive immunity treatment trials [57, 58]. This represents a potential therapeutic strategy in the treatment of AD patients who already have large amounts of Aβ deposits. Other investigators have demonstrated improved cognitive function that accompanied reduction of Aβ in AD mouse models [36, 54–56]. Taken together, these data suggest a potential therapeutic role of REMFS in human diseases, such as LOAD.

Other studies suggested that EMF exposures enhance pathways involved in Aβ degradation through upregulation of the HSF1 pathway [43], the autophagy-lysosome system [26], the ubiquitin–proteasome system [23, 25], and a reduction in β-secretase activity following REMFS thus producing a protective effect through reduction of Aβ [56]. Furthermore, REMFS also targets multiple aging and cell defense pathways that are involved in AD, including oxidative stress [19], cytoprotection [20], inflammation [27], mitochondrial enhancement, and neuronal activity [55], thereby making REMFS a potential multi-target therapeutic strategy for AD and other age related diseases.

## Proposed mechanisms of REFMS and biosystems interactions

### Low energy challenges

There are two challenges that underly an explanation of the REMFS and bio-systems interaction: (1) the low energy in these EMFs is several orders of magnitude lower than k_B_T (k_B:_ the Boltzmann constant, T: room temperature). This leads to information being transmitted via a phenomenon known as the k_B_T paradox in which information is hidden in thermal noise [59]. (2) Classical models of molecular dynamics would hold that the excited state produced by the EMF would promptly dissolve due to the thermal excitations that restart when the EMF exposure is eliminated, which is not seen [60].

REMFS exposure transmits a very low energy that is insufficient to excite electrons and is thereby considered non-ionizing. The photon energy of REMFS at 50 MHZ is 2.0678 eV^−7^, at 64 MHz it is 2.64 eV^−7^, and at 915 MHz it is 3.7841e–6 eV. Additionally, protein conformational changes cannot occur under direct electric field magnitudes lower than 10^8^ V/m [61] and REMFS only produce 16.22 V/m [62]. REMFS energies are incapable of directly causing the dissociation of chemical bonds such as the H–O–H covalent bond of a water molecule (H_2_O) because this type of reaction would require 493.4 kJ/mol or 5.1138 eV [63, 64], an exponentially higher amount of energy.

Thus, classical physics is unable to explain the biological responses to REMFS. Nevertheless, quantum physics provides an explanation of how this reaction occurs. Here we consider low energy EMF with frequencies below the THz wavelength. Interestingly, high energy EMF are not able to produce the biological effects of the low energy EMF [65]. In addition, Panagopoulos found that oscillating EMF with frequencies lower than 1.6 × 10^4^ Hz produce biological effects, even at very low intensities. Conversely, as the frequency of the EMF increases to more than 1.6 × 10^4^ Hz a higher field intensity is required to produce biological effects [66].

One plausible explanation why high EMF frequency is less likely to produce biological effects could be the reduced hydrogen bonding seen in higher temperatures which is directly correlated with higher energy [67]. In an interesting study, THz-exposed cells exhibited some biological responses such as increase in heat shock protein expression. Results suggest that the biological effects imposed by THz radiation appear to be primarily thermal in nature [68]. Conversely, the effects of the RF and microwave are primarily non-thermal, therefore suggesting a different mechanism. This difference could be due to the effects that RF and microwave range correlates to the rotation of polyatomic molecules and higher frequency correlates to the vibrations of flexible bonds [68].

Another possible explanation is the effect of the REMFS oscillation on the H-bond at the quantum level. It may be produced by the fact that the REMFS frequency is much slower (Hz to GHz) than the H-bond frequency (74 THz), thus inducing the H-bond to act as a driven quantum harmonic oscillator under REMFS exposures [69, 70] (Eq. 1) in a time-dependent adiabatic perturbation [71]. REMFS is a continuous field exposure (perturbation) which acts slowly enough to allow the quantum system sufficient time for the functional form to adapt [71] (adiabatic process), and consequently become able to cause changes in the probability density and amplitude. Under faster excitatory frequencies the driven harmonic oscillator has no time to follow the excitatory frequency like in the classical solution [72].

### Changes in water depend on EMF properties

EMF produce their effects by the electric field [67], rather than the magnetic field. Depending on the frequency and intensity of the EMF, it can change water structure through different influences. Consequently, there are some inconsistencies in the effect of EMF on the H-bond network (HBN) [73] and on the biological response. De Nino demonstrated a decreasing coherent population accompanied by the increase of the intermediate population under high amplitude of field (0.15) [74]. Conversely, Shen found stronger polarization and a higher degree of association in exposed water to low frequency EMF and low amplitude of field (0.15 T) [75]. These data suggest that the effects of EMF on H-bond and its biological effects are determined by the strength of the external field. The values and distribution of the internal fields depend on the frequency, polarization, field strength, field distribution of the external fields, the configuration of the tissue, and its dielectric properties [76].

### Potential mechanisms

There is no generally accepted mechanism to explain the role of low frequency EMF in biological systems, though multiple mechanisms have been proposed. There is inconsistency amongst these theories, which can be explained by a lack of consideration for varying energy levels, tissues, or the quantum effects of these fields on water molecules. Below are examples of these proposed mechanisms:

1. RF-EMF alters the structure of the water surrounding some biomolecules, which allows water to store and release a greater amount of energy under EMF [77]. The theory is that RF-EMF exposure induces water auto-ionization to produce hydronium, which in turn protonates biomolecules to activate biological pathways. The energy that would be generated by this proposed mechanism is so high that it would cause an increase in temperature that has not been observed in REMFS exposures.
2. Protein and protein complexes (HSF1-Hsp90) [43], as well as elements of RNA and DNA [78], are EMF sensitive and can behave as EMF-sensors that operate by disruption of their conformation to form secondary structures in response to EMF variations. Structural transitions can uncover or obscure important regions of RNA, such as binding sites, or lead to dissociation of protein complexes which can release active transcriptional factors. These changes, then, affect the translation rate of nearby protein-coding genes to activate biological pathways. It is an unlikely mechanism because we observed that the initial event in our experiments is not DNA activation; heat stress is required to initiate translation.
3. Cells (e.g., neurons) possess the ability to yield constructive interference effects that enhance their intensities at several points [79–81], so that an applied EMF could be amplified to produce conformational changes in some proteins, transcriptional factors, and RNA. This hypothesis also requires very high energy to break bonds and would cause a thermal response.
4. Second-harmonic generation increases energy of photons whereby water molecules align during EMF exposures [82]. In this mechanism two photons with the same frequency interact with a nonlinear material to create a new photon with twice the energy of the initial photons. This hypothesis is unlikely to be the mechanism because even this doubled energy of the new photon would be still too low to break any chemical bonds.
5. EMF forces affect electrons in a way that weakens H bonds. This destabilization can act on H bonds holding DNA strands together, thereby affecting transcription. The low electron affinity of the bases, which has been previously identified in electromagnetic response elements (EMREs), are needed for EM field interaction with DNA. This theory is also less likely because we demonstrated that DNA activation is not the initial event in our experiments.
6. Another hypothesis is that of a resonant frequency as the mechanism of this interaction [83]. However, the exposures are billions of folds different [78, 84–87], thereby creating a wide range of frequencies capable of causing the same biological effects. This makes the hypothesis of a resonant frequency very unlikely and difficult to substantiate.
7. High energy vibrations of ions have are less likely the cause of the RF and biological systems interaction because the mobility of ions is low among these exposures [88].

### Summary of our hypothesis

In this paper, we do not intend to produce a systematic review of all the EMF bio-effects; instead, we are trying to develop a theoretical framework for our experimental results. The mechanism postulated here explains the activation of the Heat Shock Factor1 (HSF1) via REMFS. We believe that different EMF frequencies produce different mechanisms of action on biological systems. For example, ionizing or thermal mechanisms produce the high energy required to remove electrons and break covalent bonds. Also, high (THz) frequencies produce their effects mainly by a thermal mechanism [68].

Here, we concentrate on studies performed on the low energy EMF spectrum (Hz to GHz) to describe mechanisms by which non-ionizing, non-thermal, non-modulated, continuous EM waves induce biological effects. We use results collected through our research on human cell cultures and other researchers’ recent results on mouse AD models to support these theories.

Our initial REMFS experiment used an EMF frequency of 50 MHz and a specific absorption rate (SAR) of 0.5 W/Kg. We determined that REMFS activated HSF1 in cell cultures of in lymphocytes and fibroblasts [43], increasing 70-kDa heat shock proteins (Hsp70) chaperone levels and ultimately postponing aging and death in cell cultures. Recently, we demonstrated that REMFS at 64 MHz with a SAR of 0.6 W/Kg for 14 days reduced potentially toxic amyloid-β peptide (Aβ) levels by 46% in cultures of primary human brain (PHB) when compared to non-exposed controls. A decrease of Aβ levels in PHB cultures also appeared with different duration and power protocols. Of note, Aβ precursor protein (APP) levels and non-APP processing pathway products were not altered by the treatments, suggesting enhancement of Aβ degradation as the possible mechanism of Aβ reduction.

**Fig. 1:**
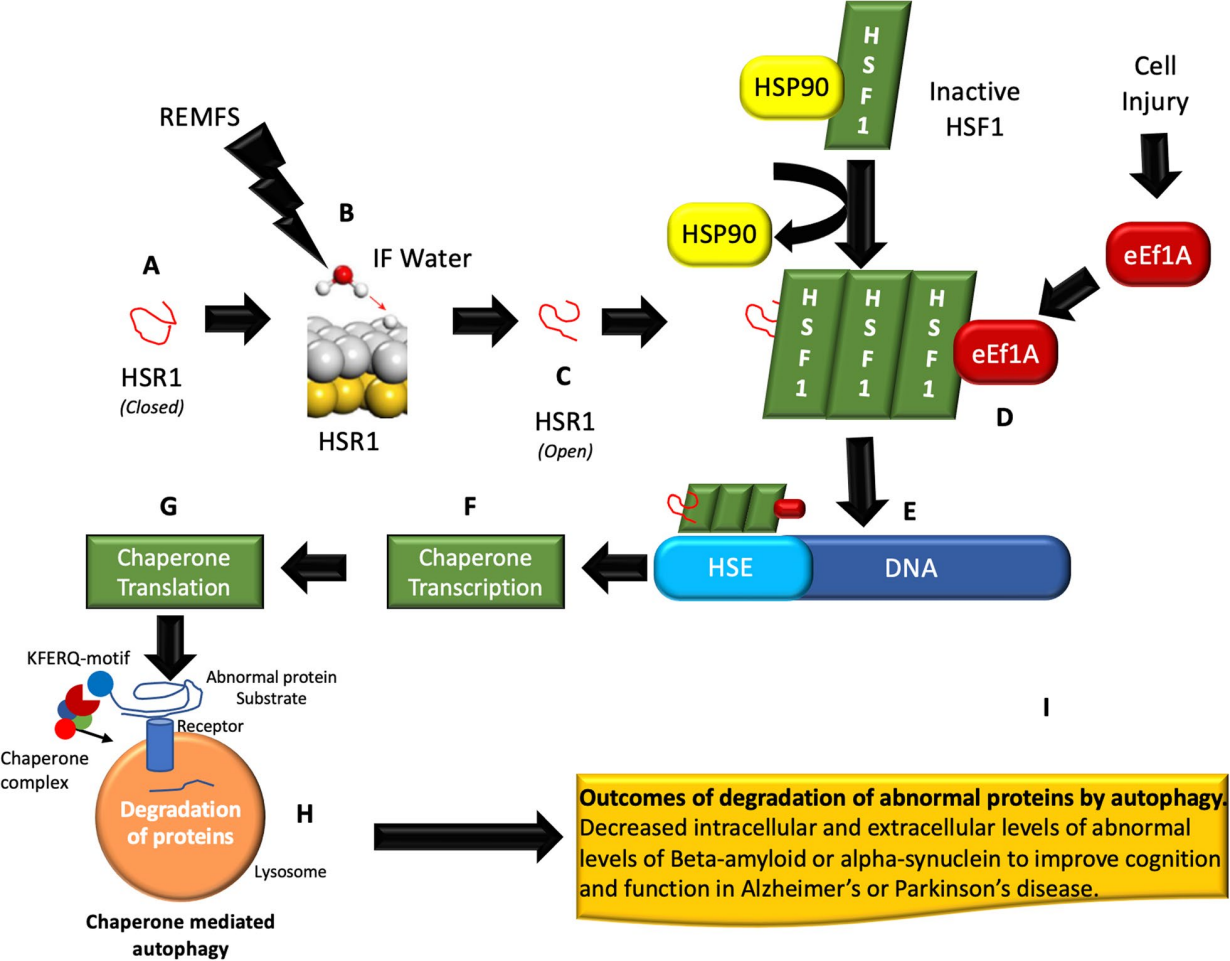
Multidimensional sequence of events of the REMFS and biological system interaction. **A** The Long noncoding RNA Heat Shock RNA1 (HSR1) in a closed conformation. **B** REMFS exposures affect the interfacial (IF) water of HSR1 to produce proton tunneling. **C** Protonation of nucleic acids of HSR1 produce an open HSR1 conformation. **D** Open HSR1 binds to Heat Shock Factor 1 (HSF1), releases HSP90, then HSF1 trimerizes and forms complex with the HSR1 and the elongation factors 1 alpha (eEF1A) after cell injury. **E** The complex binds to the Heat Shock Elements (HSE). **F** and **G** This process initiates Chaperone translation and transcription. **H** Chaperones induce autophagy and degradation of abnormal proteins. **I** Clinical outcomes of REMFS exposures

We hypothesized that a multidimensional sequence of events explains the REMFS and biological system interaction (Fig. 1) from the quantum to the molecular, cellular, and organismal levels. The REMFS mechanism is a combination of the oscillatory quantum [89] and molecular [67] effects on the interfacial water HBN surrounding biomolecules; specifically in REMFS, those H-bond’s confined to the first layer of the interfacial water in the vicinity of the non-coding RNA Heat Shock RNA-1 (HSR1) [90]. This EMF oscillation causes the H-bond to behave like a driven quantum harmonic oscillator [91], thereby increasing the amplitude of the H-bond vibration [92] within the interfacial water that naturally surrounds nucleic acids. This shortens the length of the H-bond, increasing the probability of proton tunneling [93] and protonation of the nucleic acids [94]. This leads to the formation of tautomers [95] that produce conformational changes in HSR1 [90] to allow binding and activation of HSF1. Subsequently, HSF1 binds to DNA to express chaperones that initiate chaperone autophagy and degradation of abnormal proteins such as Aβ with consequent clinical improvement in Alzheimer’s disease (AD).

The main question in the EMG and biological systems interactions is what the target of these fields is and how the target is affected to produce a response. We hypothesized REMFS oscillations on the interfacial water cause a combination of quantum and molecular vibrations responsible for the biological effects under these fields. The mechanism of the EMF and biological systems interaction could be thermal or non-thermal. Previous studies showed that REMFS acts by a non-thermal mechanism [43, 96]. A temperature-dependent (i.e. thermal) mechanism produces changes in the rates of biochemical reactions as a result of heat energy transfer to the target receptor. In contrast, nonthermal mechanisms are not associated with a change in temperature, but rather with oscillations of the RF which cause vibrational energy transfer, and ultimately a change in the REMFS receptor [97]. We will discuss the changes at the quantum and molecular levels in more detail in the following subsections.

## Exposure times and regimens

Exposure time is a very important factor to achieve an effective dose that produces biological effects. To find out possible advantages and mechanisms of REMFS, it would be much more valuable to perform experimental studies to determine the effects accumulated with time and profiles of repetition. In comparison, computational studies are limited by very short exposure times. The time exposure and repetition regimen parameters differ regarding the case under study and depend on the physical and biological conditions of the exposed target.

In the particular case of REMFS and biological pathways associated with protein degradation systems in humans and mice, we observed that the minimum exposure time to produce biological effects was between 15 and 30 min of exposure with a peak response of one hour. We also observed that the minimum regimen was 3 times per week. Marchesi found that briefer EMF exposures of 15 and 30 min did not show significant differences compared to the untreated control when measuring miR-30a expression levels. However, lengthier EMF exposures of 1 h, and to a lesser extent 3–24 h, produced biological effects.

This minimum time exposure is necessary to activate and recruit enough molecules of the HSR1 to initiate protein degradation. A repetition exposure regimen leads to maintaining high chaperone levels and degradation of abnormal proteins; otherwise, cells would continue to accumulate abnormal proteins that form during cellular metabolism [26]. There should also be a balanced process of degradation and protein synthesis, which is obtained by an intermittent regimen. There is evidence that effective exposure times can vary according to the type of cell or organism, biological pathway affected, and the physical conditions of the exposure. For example, a study showed that longer time exposures are needed to obtain the maximal biological effect. They found a minimal effect in mammalian stem cells after 2 h of EMF exposure and maximum effect after 9 h of radiation [98].

Therefore, studies that focus on the minimum exposure times (MET) and minimum exposure regimens (MER) to produce biological effects can shed light on thresholds of cell capabilities. In addition, it is more likely to reduce the complexity of the EMF interaction targets in cell cultures and organisms by lowering the exposure times and regimens, which at least reduces the overall rise in temperature.

## REMFS mechanism at the quantum level

### REMFS exposures perturbs intracellular quantum systems

The REMFS interaction with biological systems allocated to multiple layers, which themselves also radiate different EM frequencies, represents a complex subject due to the scattering problem and the many-body perturbation theory (MBPT) from layered structures [55, 99]. The challenge owes its complexity to the mathematical difficulties in describing electromagnetic interaction within intracellular structures, which can be exceedingly complex due to the extremely heterogenous nature of physical and biological processes. The interactions between multiple biomolecules, atoms, ions, rough interfaces scattering, and the applied EMF exposures poses strong limitations to the development of comprehensive models for the effects of REMFS on biological system. Herein, we will describe the REMFS receptor and biological systems interaction as a consequence of quantum confinement of the interfacial water HBN by electronegative biomolecules and the electropositive nucleus [100, 101].

### REMFS receptor

The main reason we hypothesized that there is one receptor for REMFS instead of multiple receptors for each frequency is the fact that a wide range of frequencies (Hz to GHz) can induce similar biological responses. One well recognized example of this phenomenon is observed in the heat shock response [24, 49–52]. The fact that billions of frequencies (from Hz to GHz) produce the same biological effect makes the possibility that there is a receptor for each type of electromagnetic frequency improbable. Rather, it is more likely that some common receptor mediates this effect [102]. This common receptor would have to be capable of responding to the oscillating energy of the EMF exposures within a wide range of frequencies to induce the activation of biological pathways [102]. If so, this receptor must be able to induce conformational changes, specifically biomolecules that result in a secondary structure formation that regulates transcriptional or post-transcriptional mechanisms.

Interestingly several studies have found that the interfacial water is an integral partner of biomolecules and the modulator of their activity [103]. In addition, interfacial water plays an important role in the structure of DNA and RNA, forming bridges between bases within the same strand or between two strands, and in proteins, stabilizing β-sheets and α-helices [104] In RNA, the first layer of the interfacial water is consider a part of nucleic acid structure because it defines structure, folding, and intra-molecular interactions [105, 106]. The explanation of the interfacial water as a modulator of biological activity is also proposed by Mentré. He suggested that the key properties of the interfacial water in the cell are that H-bonds are cooperative, currents of protons, osmosis, hydrostatic pressure, density variations, and selective exclusions of ions. These changes make stronger and shorter H-bonds in the interfacial water with higher heat capacity than bulk water because more energy is necessary to break its H-bonds [103].

Experimental studies have demonstrated EMF interfacial effects can produce biological effects [107–109]. Beruto et al. grew Chlorella vulgaris with and without the application of a low intensity, low frequency EMF of about 3 mT for 30 days. These exposures produce a significant effect on the exothermic clusterization step. The investigators proposed that this effect is produce by the interfacial water of glycocalyx of the external region of microalgal membrane because it can interact with chemical species present in the environment [109].

The organization of the interfacial water depends on the type of bio-surface electrostatic forces [103]. The first layer of the interfacial water is in special quantum confinement, since the polar and charged groups of the nucleic acid interact with the surrounding H bonds [110] from the interfacial water. This interaction actively affects the structure, function, and H bond of biomolecules [111]. Such polar and charged groups are sources of electric fields [112], which are very important for proton transfer [113]. This proton transfer is based on the electropositive applied electric field (EF) from REMFS (16.22 V/m) on the interfacial water and the intracellular EF rearrangements from RNA HSR1 electronegative attraction of EF (− 30 to 100 kT/e) and the electropositive EF from the cytoplasm (38 × 10^6^ V/m).

Data indicates that intracellular EF rearrangements cause an electrostatic confinement of the first layer of the interfacial water, which gives it quantum properties [114]. These interfacial water molecules are ‘pseudo-immobilized’ and, therefore, confined to sub-diffraction volume. Of special note, this electrokinetic confined or trapped water exhibits a quantum tunneling behavior [115]. These data suggest that the interfacial water is a potential target for the effects of REMFS, which is confirmed by other investigators that proposed that water is a sensor for low energy EMF [116].

### H bond under REMFS

The pico- to sub-picosecond lifetimes of H-bonds are too short for experimental techniques such as nuclear magnetic resonance (NMR) and dielectric spectroscopy time window, so it is hard to perform experiments with water under EMF [117]. However, in a recent study applying an intense THz pulse (peak electric field strength of 14.9 MV/cm) to liquid water led to increasing H-bond stretching and bending vibrations. [118]

Similar evidence that polarized REMFS radiation causes its biological effects comes from the fact that it induces the dissociation of water into its constituent elements [119]. Rao examined distilled water under a polarized 2.45 GHz exposure. The Raman spectra of the treated water showed significant changes in the O–H stretch bond, which predisposes to proton tunneling and protonation of the surrounding molecules. Interestingly, despite different experimental conditions such as frequency (THz versus Hz), most of the conclusions are consistent with the fact that very different REMFS can produce water dissociation [120].

Furthermore, EMF exposures strengthen and shorten the H-bonds on the surface of biomolecules. For example, when hemoglobin and bovine serum albumin in water solutions were exposed to 50 Hz, samples revealed a significant increase in the absorbance signal of the Amide II band and an up-shift toward the high energies after exposure. These results suggested that EMF exposures strengthened the H-bonds of the secondary structures of these proteins [121].

Although REMFS increases the rotational kinetic energy of bulk water, the effects on the water of the first layer surrounding biomolecules are most prominent. The water of the first layer in the vicinity of biomolecules has a forced orientation and cannot rotate easily. However, under REMFS, it can undergo large-amplitude librational motions [122, 123]. REMFS oscillations at the molecular level produce rotation of water molecules within the first layer of the interfacial water as they try to "flip" their polar directions to match the polarity of the radio wave radiation. As a result, the oscillating electric field from the REMFS forces the water dipole moments to reorient themselves [67], which affects the H-bond that connects the first layer of the interfacial water.

This effect can be observed in biological tissues, where all polar molecules, such as water, are forced to oscillate in phase with the field and on planes parallel to its polarization [66, 124] This is one of the most important factors for the quantum effects of REMFS: the man-made polarization of the excitatory oscillation of REMFS on contact with the interfacial water of the bio-system. These oscillations have a lower frequency relative to the exposed quantum system. They will change the frequency of the system to the excitatory frequency [65] like a driven harmonic oscillator [91, 125]. The system is driven by energy imparted upon the harmonic oscillator continuously by an external force [126]. If the excitatory frequencies are slower, the oscillator frequency is pulled towards the excitatory frequency [126].

### REMFS produces proton tunneling

Proton tunneling is a type of quantum tunneling that causes the transfer of a proton in one site to the closest site isolated by a potential barrier. Proton tunneling is commonly related to H-bonds. The hydrogen atoms are linked to two non-hydrogen atoms via an H-bond at one end and a covalent bond at the other.

H-bonds are classified based on energy or on geometry [127]. Several studies have identified the transitions from weak to moderate to strong H-bonds and the physical bases of the main geometry-based H-bond strength classifications. In this study, we use the geometric classification where the hydrogen bond is very strong when the distance between donor and acceptor atoms is in the range 2.2–2.5 Å, strong if it is in the range 2.5–3.2 Å, and weak if it is in the range 3.2–4.0 Å [128].

As mentioned above EMF decrease the water H-bond length and increase the H-bond angles as a function of the large amplitude motions [129] (Eq. 2). These large amplitude vibrations decrease the H-bond length of the first layer of the interfacial water to the oxygen of the nucleic acid (O−H···O) to values below 1.85 Å [130], which are ideal lengths for rising the probability of proton tunneling [131, 132]. This effect depends on the spatial location of the molecule with respect to the field [133], affecting the HBN [134] in an anisotropic manner. Thus, as the front water molecule is rotated [135] and hydrogen pairs with net dipole moment, the new configuration changes the hydrogen-bonding energy and distance. This water reorientation is of importance in multiple quantum processes, including proton transfer [136], proton transport [137, 138], and hydration of RNA or proteins for their function [139].

The amplitude of the H-bond varied between 0.18 and 0.22 Å for liquid water at 25 °C [129, 140]. In ab initio calculations large-amplitude motions caused by EMF exposures affect the bond distances and decrease the barrier distance from the H of the interfacial water to the O from the carbonyl group of the nucleic acid, consequently increasing the probability of proton tunneling [141] (Eq. 3).

Evidence that EMF cause tunneling is shown in a combined experiment and computer simulation demonstrated that the Hydrogen–Oxygen (H···O) distance is critical for tunneling and the rotation of the hydrogen from the confined water toward the O modulates the H···O distance [142]. Transitions arising from both pure rotation and rotation-tunneling can occur [143]. These data indicate thermodynamically balanced motions that control the donor–acceptor distance and site of active electrostatics, developing conformations apt for proton tunneling [144].

Additionally, a mathematical model found that changing the H-bond length by the radius of a hydrogen atom (0.05 nm) changes the transmission coefficient or tunneling current by 210%, suggesting an extreme sensitivity of tunneling to distance changes on the scale of atomic dimensions [145]. Furthermore, dynamical complexity increases with the exposure to the REMFS frequency during barrier penetration of the tunneling process [141]. For example, studies have found evidence for Coherent Proton Tunneling in a HBN at a tunneling frequency of 35 MHz, a frequency somewhat close to the REMFS exposures (64 MHz) used in our experiments [146].

Further evidence that supports tunneling in REMFS is a quantum calculation study that found that the flipping processes of water under quasi-one-dimensional (1D) confinement produces quantum tunneling effects [147]. The confinement in this study is very similar to the electrostatic confinement of the interfacial water of the HSR1 mentioned above during REMFS exposures.

In addition, several studies have found that EMF cause proton tunneling to produce tautomers in nucleic acids. Cerón-Carrasco found that electric fields induce proton transfer which produce tautomers in nucleic acids using Quantum Mechanical (QM) calculations [148]. The electric field decreased the potential barrier leading to the tautomer by 20–55 kJ mol^−1^. The study concluded that in the presence of EFs, only Guanine-Cytosine fit the necessary kinetic criteria to be considered a viable route to formation of tautomers.

In a more recent study Cerón-Carrasco investigated a more accurate DNA fragment in a simulation. The study found that at higher electric fields, tautomers are more stable than canonical bases [93]. In a classical molecular dynamics study, Cerón-Carrasco found that a continuous electric field exposure produces conformational changes in nucleic acids in 10 picoseconds [149].

Furthermore, Gheorghiu found that EM effects occur when the electric field is parallel to the H-bond axis [150]. Parallel electric fields were found to have a great influence on the energetics of the Guanine-Cytosine proton transfer tautomerism. It is important to consider that these effects occur under high electric fields like the found in the intracellular interfacial water of the HSR1 as mentioned above.

All these data suggest that REMFS promotes proton tunneling by oscillations that increase the amplitude of the H-bond vibrations of the interfacial water and modulate proton-acceptor distance, which increases the probability of tunneling proportional to the amplitude and the proton-acceptor distance [141, 151]. This protonation creates tautomers in RNA and DNA that affect biological changes.

### Mathematical model

**Fig. 2:**
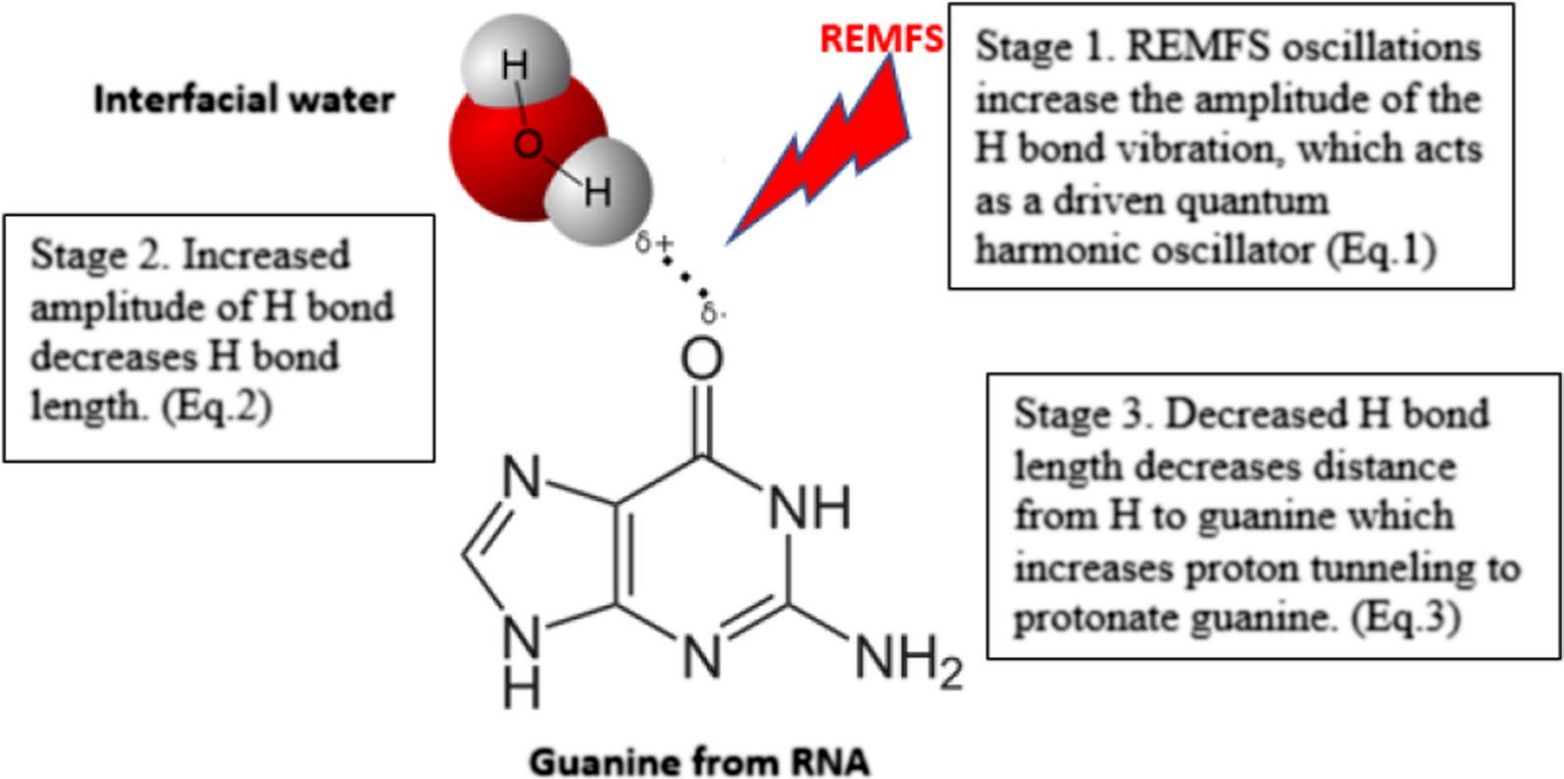
REMFS quantum effects on the first layer of the interfacial water of RNA. REMFS (repeated electromagnetic field stimulation)

**Fig. 3:**
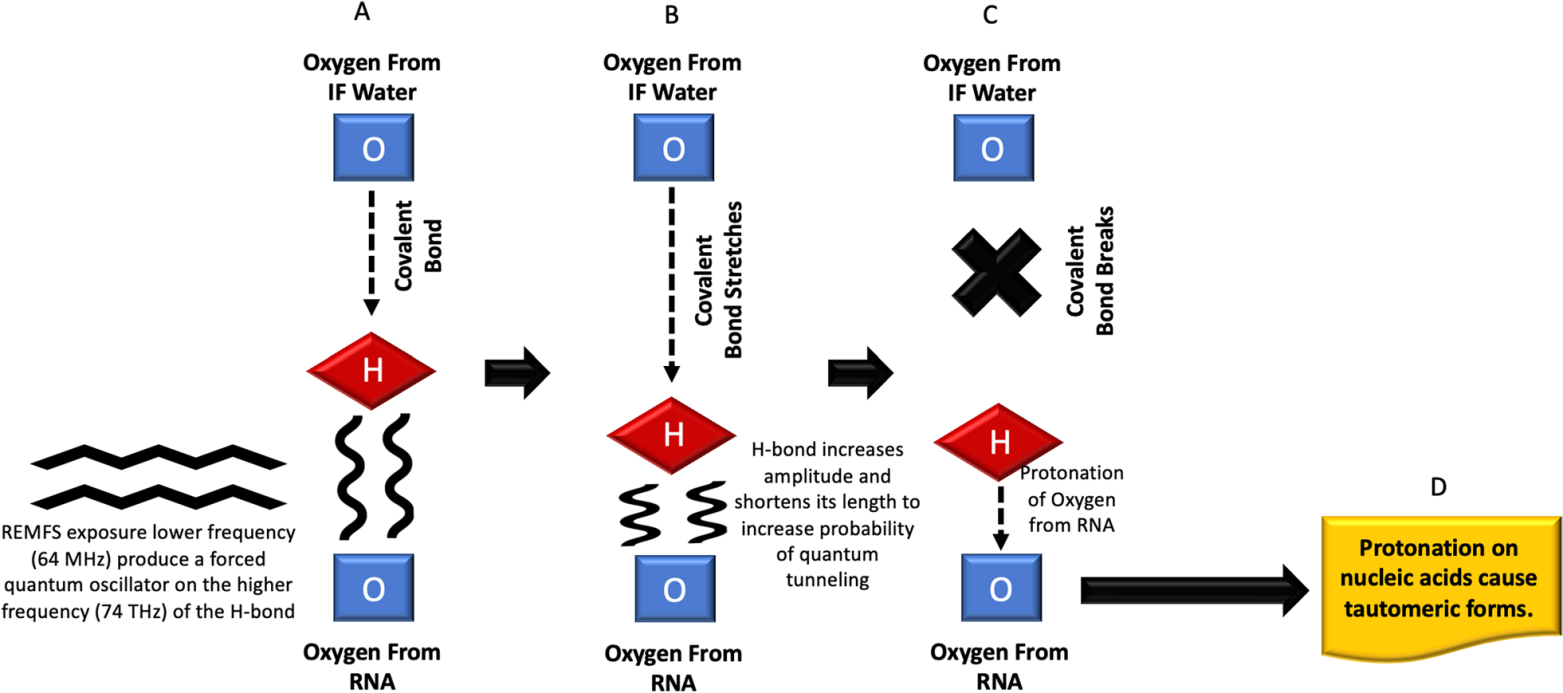
Quantum proton tunneling under REMFS Stage 1. **A** REMFS oscillations affect the H-bond and produce a driven quantum harmonic oscillator which adapts to the REMFS frequency (higher frequencies do not give enough time to allow the system to adapt, they also produce thermal effects). **B** The Interfacial (IF) water H-bond length shortens the distance from acceptor oxygen from RNA and water covalent bond stretches. **C** Covalent bond breaks and Hydrogen protonates acceptor oxygen by proton tunneling. **D** Protonation cause tautomeric forms

Our challenge was how to explain why a low energy wave causes biological effects. Therefore, we hypothesized the quantum effects of REMFS. We established a numerical model for the interaction between the REMFS exposures and biological systems at the quantum level [152]. For simplicity, we divided them into three stages, each with its equation (see Fig. 2):

*Stage 1.* REMFS vibrating energy produces a time-dependent adiabatic perturbation on the H-bond of the first layer of the interfacial water (FLIFW) [152]. During the REMFS exposures that increase the amplitude of the H-bond vibrations, the H-bond of the FLIFW changes state to a driven quantum harmonic oscillator. REMFS affects the H-bond situated near the oxygen (O) of the guanine of the RNA (GRNA). The following formula (Eq. 1) estimates the amplitude increment of H-bond oscillation as a driven quantum harmonic oscillator (see Fig. 3) system under REMFS [70].

*Equation 1:*

First, we obtain the time dependent periodic force:

1.1 $$\mathrm{F}(\mathrm{t})=\frac{\mathrm{A cos}(\mathrm{\omega t }+\mathrm{\varphi })}{\sqrt{2\mathrm{m\hbar }{\upomega }_{0}}}$$

Then we obtain the amplitude:

1.2 $$\mathrm{A}=\frac{\mathrm{F }\left(\mathrm{t}\right)\sqrt{2\mathrm{m\hbar }{\upomega }_{0}}}{\mathrm{cos}(\mathrm{\omega t }+\mathrm{\varphi })}$$

where: A = amplitude, ω = vibration frequency of the periodic force, ${\upomega }_{0}$ = frequency of the oscillator, m = mass of the oscillator, φ = phase of the driving field, t = time of the exposure, F (t) = time dependent periodic force, and ħ = reduced Planck’s constant. [70]

In our experiments, F = q (E + v x B), q = 1.062 × 10^–19^ C (proton charge), v = 3 × 10^8^ m/*sec*, E = 17 V/m = 17 N/C, B = 3.89 × 10^2^ A/m = 146.2 × 10^3^ V/m = 146.2 × 10^3^ N/C, m = 1.673 × 10^–27^ k (proton mass), ħ = 1.054 × 10^–34^ J/s, t = 3,600 s, $\mathrm{\varphi }=0$, ω = 64 MHz, and ω_0_ = 74 THz.

*Stage 2.* The value of the calculated H–O distance is very short (1.85 Å) [153]. REMFS shortens the distance between H of the FLIFW to O of the GRNA by increasing amplitude of the H-bond vibration. The change in the H-bond distance as a function of the amplitude of the oscillation is calculated in Eq. 2 [129].

*Equation 2:*

First, we find the average over the inter-nuclear configurations of the interfacial water and RNA nucleic acids:

2.1 $${\mathrm{r}}_{\mathrm{a }}^{\mathrm{exp}}= \langle \mathrm{r}\rangle = \int {\mathrm{F}}_{\mathrm{r}}\left(\mathrm{q}\right)\mathrm{ P}\left(\mathrm{q}\right)\mathrm{d q}$$

where: ${\mathrm{r}}_{\mathrm{a }}^{\mathrm{exp}}$ = the average over the inter-nuclear configurations of the interfacial water and RNA nucleic acids, F_r_ (q) = Variation of Bond distance, P(q) = Probability function.

The equation of P (q) in the classical Boltzmann approximation is:

2.2 $$\mathrm{P}\left(\mathrm{q}\right)=1/\mathrm{N}\int \mathrm{exp}\left(-\frac{\mathrm{V}\left(\mathrm{q}\right)}{\mathrm{RT}}\right)\mathrm{dq}$$

where: V(q) = Potential function of floppy motion of the molecule, N = Normalization constant, q = amplitude coordinate of the oscillations.

Then, the dynamical correction term ${\updelta }_{\mathrm{dyn}}$ is:

2.3 $${\updelta }_{\mathrm{dyn}}={\mathrm{F}}_{\mathrm{r}}\left({\mathrm{q}}_{\mathrm{min}}\right)-{\mathrm{r}}_{\mathrm{a}}^{\mathrm{exp}}$$

where: ${\mathrm{r}}_{\mathrm{a}}^{\mathrm{exp}}$= operational parameter determined from the least squares fit to the experimental electron diffraction intensity curves. ${\mathrm{F}}_{\mathrm{r}}\left({\mathrm{q}}_{\mathrm{min}}\right)$ = bond distance at the minimum of the potential function.

This equation predicts the shortening of the H-bond of the interfacial water under the time dependent perturbation caused by REMFS.

*Stage 3.* REMFS oscillation increases the amplitude of the H bond and decreases distance of the H from the FLIFW to the O from the GRNA, predisposing for H tunneling [70]. The probability of tunneling is proportional to the square of the amplitude. The barrier thickness or the decreased distance estimates the quantum tunneling probability by Eq. (3) [141].

*Equation 3:*

3.1 $$-\frac{{\mathrm{\hslash }}^{2}}{2\mathrm{m}}\frac{{\mathrm{d}}^{2}\Psi (\mathrm{x})}{{\mathrm{dx}}^{2}}=(\mathrm{E}-{\mathrm{U}}_{0})\Psi (\mathrm{x})$$

where: E < U_0_.

Hydrogen energy

Energy of the incoming particle/ Hydrogen energy € = 0.306 eV, mass (m) = 1.673 × 10^–27^ kg, height of barrier (U_0_) = 5.099 eV, thickness (d) = 2.6 nm, attenuation factor (α) = 0.475678 × 10.^12^ l/m, ratio of the exit and incident amplitudes = 0.999952433311635

3.2 $$\Psi ={\mathrm{Ae}}^{-\mathrm{\alpha x}},\mathrm{ where \alpha }=\sqrt{\frac{2\mathrm{m}({\mathrm{U}}_{0}-\mathrm{E})}{{\mathrm{\hslash }}^{2}}}$$

where: E < U_0_.

Energy of the incoming particle/Hydrogen energy (E) = 0.306 eV, mass (m) = 1.673 × 10^–27^ kg, height of barrier (U_0_) = 5.099 eV, thickness (d) = 2.6 nm, attenuation factor (α) = 0.475678 × 10 ^12^ l/m, ratio of the exit and incident amplitudes = 0.999952433311635, This equation finds the probability of proton tunneling.

These three stages and their respective equations have allowed us to develop a numerical model that can predict why the time dependent perturbation produced by REMFS alter the H-bond of the water of first layer that surrounds biomolecules. This H-bond acts as a quantum damped harmonic oscillator to increase the probability of tunneling and allows for the protonation of the nucleic acids of the surrounding RNA that in turn activates biological pathways.

## REMFS mechanism at the molecular level

### Nucleic acids tautomers cause conformational changes in RNA and DNA

In RNA, nucleic acid bases occur in several tautomeric forms due to protonation of the nucleobases [95]. Tautomers [154] are used by multiple RNA to produce their functions [95, 155].

Furthermore, it is well known that tautomeric equilibria are affected by several chemical and physical factors such as metals, temperature [156], pH [156] and recently EMF exposures [93, 157]. Also, tautomer inter-conversions can adopt various secondary structures responsible for a variety of functions during biological processes like RNA conformational changes, DNA replication, packaging, and transcription [158, 159]. Often, such conformational changes promote binding to activating factors that in turn affect transcription and translation of proteins.

Tautomerism causes conformational changes in the catalysis of self-cleaving RNA [95] to produce a wide array of biological functions [160–162]. The importance of tautomerism in the function of a biomolecule is highlighted in all crystal structure changes of TPP dependent riboswitches. TPP is in an extended conformation, with the orientation of the thiazolium and pyrimidine rings within hydrogen-bonding distance [163]. Here, a glutamic acid protonates and stabilizes the imino tautomer of the pyrimidine ring to lead to the ylide conformation bound in either the TPP-dependent riboswitch or a TPP-dependent enzyme [164].

Investigators using quantum chemical computations detected all possible protonated base pairs in RNA crystal structures. Data showed 18 different protonated base pair combinations from RNA and proposed a theoretical model for base pair combination [165].

Guanine and cytosine protonation affects RNA structure and function. An interesting QM analyses suggested that the guanine protonation can be a crucial factor in structure and function of RNAs [166]. The different RNA structures come from the changes of single and double bonds in the ring systems of purines and pyrimidines [167]. In a theoretical study, Chaudry calculates the quantum source of the tautomerism in DNA [168]. This tautomerism shows enhancement under EMF exposures.

Evidence suggests that REMFS protonates biomolecules [60], resulting in important tautomeric interconversions and conformational changes [95, 169]. Experimental and theoretical studies show that externally applied electric and EMF produce biologically relevant tautomers and conformational changes in RNA. An experimental study showed the effects of electric fields on RNA conformation changes and orientation by ultraviolet absorbance and electric dichroism [170]. In another study they found long-lived conformational changes in RNA by electrical impulses. The researchers applied an electric field of about 20 kV/cm to induce large dipole moments by shifting the ionic atmosphere, which caused strand repulsion and conformational changes in the RNA. Interestingly, the fields used were of the same intensity as those found in nerve transmission.

Furthermore, other biomolecule structures are affected by REMFS. De Ninno observed structural changes in Glutamic acid induced by exposure of REMFS at 50 Hz. The IR spectra contained stretching and bending bands of the protonated COOH which is attributable to the coupled C-O stretch and O–H bend of the COOH group [171]. Another study determined the tautomeric protonation of N-methyl piperazine. They performed theoretical calculations and practical experiments. These results suggest that proton relocation occurs by solvent assistance in water or proton jump. They found that predicted activation free energy was about 10 kcal/mol based on variable temperature nuclear magnetic resonance experiments [172].

All these data suggest that REMFS can cause tautomerism and conformational changes in RNA. This mechanism is similar to the regulation of HSR by RNA thermometers [173] in bacteria [174].

### REMFS activates HSF1 and chaperone expression

REMFS, heat, alcohol, hypoxia, metal ions, peroxide, amino acid analogs, and other stressors activate HSF1 and the HSR [175]. Most of these factors cause denaturation and accumulation of abnormal proteins, which induce the HSR [176, 177]. However, REMFS exposures are not likely to produce protein denaturation, so the mechanism must be related to an EMF-sensitive biomolecule such as HSR1. EMF exposure also increases HSF1-heat shock element binding activity, thereby directly contributing to the activation of HSF1 and the stress-induced Hsp70 [175] transcription and translation in cells exposed to REMFS [178, 179].

HSF1 is a transcriptional factor that is a master regulator of stress gene expression (molecular chaperones) [180]. Recently, in addition to chaperone expression, accumulating evidence indicates multiple additional functions for HSF1 beyond chaperone production. HSF1 acts in diverse stress-induced cellular processes and molecular mechanisms, including the endoplasmic reticulum, unfolded protein response and ubiquitin–proteasome system, multidrug resistance, autophagy, apoptosis, immune response, cell growth arrest, differentiation underlying developmental diapause, chromatin remodeling, cancer development, and aging [181].

REMFS produces biological effects through HSR1 [182] which activates HSF1. HSR1 employs a similar mechanism as that of bacterial RNA thermometers to sense temperature and energy changes in the cell and ultimately regulate the translational machinery [183]. HSR1 is a long non-coding RNA that undergoes conformational changes from a close to an open structure under thermal radiation exposure (THz to GHz frequencies). These conformational changes in HSR1 are required for the binding and activation of HSF1 [90]. Computer simulations reveal that HSR1 is composed of an extensive secondary structure that changes predictably within a physiological range of temperatures [90] and EMF exposures without heating [184].

Another important co-factor in the activation of HSF1 is the translation elongation factor (eEF1A), which is a key component regulating the actin cytoskeleton architecture in the cell [185]. A full HSF1 activation requires a combination of purified HSR1 and eEF1A in-vitro at physiological concentrations [186]. Under normal conditions HSR1 is present in an inactive “closed” conformation. During heat shock or EMF exposures, HSR1 “switches” to the “open” conformation that activates HSF1 and releases it from its repressor Hsp90, while after a stress a massive release of eEF1A from cytoskeleton collapse (from misfolded proteins) can then fully activate the newly freed HSF1 [90]. In contrast, under REMFS exposure alone, there is no cytoskeleton collapse [43]. The role of REMFS in this process is to promote binding of HSR1 to HSF1, with a subsequent release of HSF1 from its repressor Hsp90 [43].

Triggering the HSR by stressors after REMFS treatment produces a fast and vigorous expression of Heat shock proteins (Hsps) [187]. Protein aggregation is an important factor in the progression of aging and age-related diseases such as AD [188]. Several pathways are associated with abnormal protein clearance, including molecular chaperones, the ubiquitin–proteasome system, and autophagy pathways [189]. The production of these chaperones depends on the activation of HSF1, an event attenuated by the aging process [190]. HSF1 is repressed by the Hsp90 complex and released to get activated under several cellular stresses [191].

Similarly, REMFS exposure also releases HSF1 from the Hsp90 complex [43]. Once released from the Hsp90 complex, it trimerizes spontaneously to bind DNA, an event that produce increased amounts of Heat shock proteins such as Hsp70, Hsp90, etc. Once chaperones are produced, they bind abnormal proteins. Excess Hsp90 binds to HSF1 trimers and causes them to dissociate and revert once again to the inactive, monomeric state [192].

The HSF1 effects on Hsps play a role in aging [193] and protein accumulation diseases [188]. The role of HSF1 in the aging process and age-related diseases such as AD suggests a deeper relationship between the molecular mechanisms of these two processes. HSF1 activation prevents the decline in proteostasis, the primary contributor to aging, thus delaying the aging process [39, 194, 195]. This suggests a potential role for HSF1-based therapeutic tools, such as REMFS, in the treatment of a wide array of age-related diseases [196].

Hence, it is here where molecular chaperones such as Hsp70 take on an essential role by deterring protein aggregation [197]. Two protein degradation pathways, macroautophagy and chaperone-mediated autophagy (CMA) [198], undergo age-dependent decline probably subsequent to the age-related attenuation of the HSF1 [199, 200], which is an early molecular event in the aging process [201].

### REMFS upregulates HSF1 to promote Aβ autophagy

REMFS studies have demonstrated that repeated exposures increase Hsp70 levels by activation of HSF1 [6, 43] and autophagy [26]. Additionally, HSF1 upregulates ATG7 and RIPK1 to promote autophagy [202, 203]. Several studies have suggested that heat shock proteins prevent protein accumulation such as amyloid deposition [204–207]. Genetic overexpression of HSF1, reduces Aβ levels, induces autophagy, and up-regulates production of chaperones. Increased expression of HSF1 ameliorates AD-like cognitive deficits in PDAPP transgenic mice, which produce excess levels of human APP [208]. Furthermore, Hsp70 promotes degradation and inhibits accumulation of amyloid [204, 209–211]. Hsp70 decreased Aβ levels when given to microglia from rats [212]. Hsps binds to APP and decreases the levels of Aβ40 and Aβ42 [213]. 78-kDa glucose-regulated protein (GRP78) is another member of the HSP70 family with a role in AD. In a HEK cell model co-transfected with APP and GRP78, this Hsp70 binds to APP in the ER, preventing the β/γ-secretase cleavage necessary to produce Aβ, thereby decreasing Aβ intracellular toxicity [214]. In addition, the overexpression of GRP78 decreases the level of Aβ40 and Aβ42 in mutant APP (APPsw) cells [214].

Hsp70 binds to APP by the KFERQ motif (see Fig. 1). HSP70 transports APP to lysosomes for CMA or endosomal microautophagy (eMI) for degradation to reduce Aβ oligomers levels [215]. Additionally, many pathogenic proteins including tau, α-synuclein, and huntingtin are degraded by CMA [216–218]. Hsps binds to these proteins and degrades them through the CMA or proteasome system [219]. In an interesting study, they modified Aβ as a substrate for CMA and eMI (termed as Hsc70-based autophagy) by tagging its oligomers with multiple CMA motifs. This method significantly reduced Aβ oligomers in induced pluripotent stem cell (iPSC), which are cortical neurons derived from AD patient fibroblasts [220].

Hsp70 also suppresses oligomerization of Aβ by binding to the hydrophobic region to modify their conformation [219]. Structural changes in oligomers occurred when Hsps interacted with oligomers and fibrils. However, Hsps did not cause any direct effect on fibrils, suggesting that Hsps suppress the early stages of self-assembly [221–224]. Taken together these studies confirm that the activation of HSF1 and subsequent increase in chaperone levels, especially Hsp70, by EMF exposures influence Aβ degradation pathways [225–227] by autophagy [228, 229] to lower Aβ levels.

### REMFS decreases Aβ levels in primary human brain cultures

We recently utilized REMFS to lower Aβ levels in cell cultures of primary human mixed brain (PHB). REMFS treatment decreased Aβ40 and Aβ42 levels without evidence of toxicity. Treatment started on day 7 in vitro (DIV 7). After 14 days of REMFS, we measured levels of Aβ40 peptide in exposed and non-exposed cells. The REMFS parameters were frequency of 64 MHz with a SAR of 0.6 W/Kg for 1 h daily; this treatment achieved a 46% reduction in Aβ40 levels (p = 0.001, g = 0.798), compared to the non-treated cultures. The same REMFS parameters achieved a 36% decrease in Aβ42 levels. Subsequently, we demonstrated that REMFS at 64 or 100 MHz with a lower SAR of 0.4 W/kg for 14 days achieved a comparable reduction in Aβ40 and Aβ42 levels. Furthermore, when we increased the exposure time from 1 to 2 h, there was a similar reduction in the Aβ levels. Additionally, when we increased the frequency from 64 to 100 MHz, we found a comparable difference in Aβ levels. The results of our experiments suggest that REMFS at 64 MHz with a SAR of 0.4 W/kg for 1 h (typical of that already utilized in clinical MRI contexts) would be the minimum energy needed to produce bio-effects in human neurons, specifically a reduction in levels of toxic Aβ peptides.

## REMFS safety

In contrast to our experiments some studies have shown possible harmful effects of REMFS on biological systems which resulted from longer exposure times (minutes vs days), higher power (0.5 vs 5 W/m) and higher SAR (0.5 vs.10 W/kg) [230]. The energy produced by REMFS is extremely low, making it unlikely that our studied REMFS exposures would lead to adverse health effects, especially as we did not observe any evidence of cellular toxicity or morphological changes in our cell culture experiments [43]. Furthermore, long-term mouse AD experiments (daily for up to 9 months) and a recent phase 1 clinical trial with REMFS were safe, with no toxic effects observed on multiple safety factors evaluated. Specifically, there were no toxic effects on brain oxidative stress, brain histology, brain heating, DNA in circulating blood cells, and changes in peripheral tissues [35, 36, 55]. Additionally, our experiments use the same REMFS frequency that has been used by MRI machines for decades. Since their implementation for clinical imaging, MRI exposures have had no demonstrable negative health impacts [231]. Lastly, a recent phase one clinical trial in AD did not find any behavioral side-effect, pain, tumor growth, hemorrhage, or abnormal physiological responses after 2 months of treatment with REMFS.

## Future perspectives

Any organ that shows functional decline, including the brain, kidneys, joints, liver, or heart, may benefit from engineered REMFS exposures to induce protein disaggregation by activation of the HSF1 pathway and autophagy. Therefore, we will initiate human head exposure to treat the protein aggregation caused by AD**.** The major technical difficulties for developing an exposure system are the human head geometry, the multiple tissue layers of the head, and development of an antenna that produce a homogeneous SAR on the whole brain.

Therefore, before clinical trials are considered we must determine the best electromagnetic settings for human exposures such as power output, power deposition, far field, antenna type, distance from antenna, electric field, magnetic field, etc. that will produce homogeneous internal fields when applied to a human brain with a target SAR of 0.4–0.9 W/kg. We will start with determining by mathematical and computer modeling the REMFS exposures in our biological studies that deliver safe thermal and SAR measurements to the human head [62].

Using these results, we will develop a virtual exposure system by numerical model and computer simulation. We will design a virtual antenna that delivers a SAR of around 0.6 W/kg to a simulated phantom of a human brain. With these simulations we will find the REMFS parameters that will deliver a homogeneous radiation to a human head in clinical trials [232]. In the near future, we will experimentally confirm these results using an appropriate practical antenna to expose a Specific Anthropomorphic Mannequin (SAM) human head phantom [233] with internal and external probes oriented vertically to determine the EMF parameters that will provide an effective and safe SAR for future AD treatment. Data suggest that the ideal environment for these treatments should be an anechoic chamber to prevent RF wave reflections and provide a uniform exposure to the subjects [234]. The final step will be to initiate phase 1 clinical trials in patient with early AD to determine safety and efficacy of this new potential therapeutic strategy.

## Conclusion

The current study proposes a multidimensional mechanism at the quantum, molecular, cellular, and organismal level inside a theoretical framework that may explain the results of our experiments, and those of other investigators. The proposed quantum tunneling mechanism here is the first to provide an explanation of how low energy radio-frequency radiation may induce a biological response. Quantum tunneling allows for an understanding of events occurring between single photons and biomolecules that would otherwise be extremely difficult to visualize in experimental studies. Hence, it is by way of quantum tunneling that we finally understand the intimate relationship between REMFS and the HBN of the interfacial water of biomolecules. The process is a time dependent adiabatic perturbation of the HBN that is set into motion as a photon carried along an EM wave (with a frequency lower than the H bond frequency) that forces the H bond to change its frequency to that of the EM wave, thereby increasing the amplitude of the H bond vibrations in a process similar to a driven quantum oscillator [42]. The increased amplitude will decrease the H bond donor–acceptor distance and result in an increased probability of proton tunneling [235]. Consequently, interfacial water will donate its hydrogen toward protonation of nucleic acids, and the tautomeric interconversions that ensue result in structural changes in biomolecules and RNA, namely HSR1. The secondary structure produced will then bind to HSF1 and cause its dissociation from the multi-chaperone complex, freeing it from inhibition. Once activated, the HSF1 monomer undergoes trimerization and accumulation, inducing the expression of Hsp70 and thereby activating Aβ-clearance pathways to delay cellular senescence.

Data suggests HSR attenuation goes hand-in-hand with aging, and may even be the initial event in the aging process [201], presumably due to a decrease in HSF1-DNA binding [199]. Hence, the failure of proteostasis associated with aging may be the initial event in the development of AD [39]. Consequently, it is plausible that a treatment that enhances or restores HSF1-DNA binding would improve the loss of the proteostasis observed with aging. Therapeutic implications could also be expanded to involve other age-related diseases associated with protein accumulation, such as AD, PD, LBD, and frontotemporal diseases.

The theory herein comprises an important framework that lays the foundation for understanding the interactions between EMF and organisms and provides a valuable contribution to the foundational principles that should underlie any discussion on the biological effects of EMF stimulation. Importantly, the results of our literature review and research also point to the capacity of REMFS to influence various networks within known biological systems dysregulated in AD. The potential implications of REMFS as a therapeutic modality are likely to be far in the future, but the ability of RF-EMF to significantly reduce Aβ40 and Aβ42 levels in human neurons, coupled with animal model results, indicate a pathway worth further exploration. These results in cell and animal systems are likely achieved through a combination of efficient Aβ degradation, autophagy-lysosome system [26], and proteasome system activation [23], as well as the reduction of β-secretase activity [56]. Yet, we must note that quantum tunneling-based model could explain the conformational changes of molecules involved in other biological pathways not mentioned here.

The use of REMFS as a non-invasive therapy for the management of AD holds promise and the results of a recent phase 1 clinical trial confirm its safety in humans [236]. Nevertheless, it remains necessary to take into account the complex network of genetic [237–239] and epigenetic [240] effects occurring under REMFS. As regulation pathways triggered by REMFS have yet to be clearly elucidated, current knowledge of EMF-biological systems interaction and possible adverse effects remain limited. Quantum and classical molecular computer simulations, complemented by in vitro and in vivo laboratory studies as well as clinical trials, are needed to investigate the initial and late effects of REMFS. These studies will help develop the conditions useful for its therapeutic use while avoiding any possible adverse effects. Finally, there is a need to perform mathematical modeling and computer simulation that elucidates the appropriate EMF settings for human treatments [62, 232]. Regardless, the theories that we have proposed provide the framework for observed outcomes in several cellular and animal studies that prove the potential therapeutic implications of REMFS on age-related diseases in humans.

## Data Availability

Not applicable.
