# Correction to: Multidimensional insights into the repeated electromagnetic field stimulation and biosystems interaction in aging and age-related diseases

**DOI:** 10.1186/s12929-022-00836-9

**Published:** 2022-09-09

**Authors:** Felipe P. Perez, Joseph P. Bandeira, Cristina N. Perez Chumbiauca, Debomoy K. Lahiri, Jorge Morisaki, Maher Rizkalla

**Affiliations:** 1 Indiana University School of Medicine, Indianapolis, IN USA; 2 Division of General Internal Medicine and Geriatrics, Department of Medicine, Indiana University School of Medicine, Indianapolis, IN USA; 3 Division of Rheumatology, Department of Medicine, Indiana University School of Medicine, Indianapolis, IN USA; 4 Department of Psychiatry, Institute of Psychiatric Research, Neuroscience Research Center, Indiana University School of Medicine, Indianapolis, IN USA; 5 Department of Medical and Molecular Genetics, Indiana University School of Medicine, Indianapolis, IN USA; 6 Department of Bioengineering, University of Illinois at Chicago, Chicago, IL USA; 7 Department of Electrical and Computer Engineering, Indiana University-Purdue University, Indianapolis, IN USA

## Correction to: Journal of Biomedical Science (2022) 29:39 https://doi.org/10.1186/s12929-022-00825-y

After publication of this article [1], it was brought to our attention that two references were missed in the article, the missing references are shown below:

- [28] Perez FP, Zhou X, Morisaki J, Jurivich D. Electromagnetic field therapy delays cellular senescence and death by enhancement of the heat shock response. Exp Gerontol. 2008;43(4):307–16.
- [29] Perez FP, Maloney B, Chopra N, Morisaki JJ, Lahiri DK. Repeated electromagnetic field stimulation lowers amyloid[1]β peptide levels in primary human mixed brain tissue cultures. Sci Rep. 2021;11(1):1–3.

The original publication [1] has been corrected.
